# Vitamin D Supplementation: A Review of the Evidence Arguing for a Daily Dose of 2000 International Units (50 µg) of Vitamin D for Adults in the General Population

**DOI:** 10.3390/nu16030391

**Published:** 2024-01-29

**Authors:** Pawel Pludowski, William B. Grant, Spyridon N. Karras, Armin Zittermann, Stefan Pilz

**Affiliations:** 1 Department of Clinical Biochemistry, The Children’s Memorial Health Institute, 04-730 Warsaw, Poland; p.pludowski@ipczd.pl; 2 Sunlight, Nutrition, and Health Research Center, P.O. Box 641603, San Francisco, CA 94164-1603, USA; wbgrant@infionline.net; 3 Laboratory of Biological Chemistry, Medical School, Aristotle University, 54636 Thessaloniki, Greece; karraspiros@yahoo.gr; 4 Clinic for Thoracic and Cardiovascular Surgery, Herz- und Diabeteszentrum Nordrhein-Westfalen (NRW), Ruhr University Bochum, 32545 Bad Oeynhausen, Germany; azittermann@hdz-nrw.de; 5 Department of Internal Medicine, Division of Endocrinology and Diabetology, Medical University of Graz, Auenbruggerplatz 15, 8036 Graz, Austria

**Keywords:** vitamin D, guideline, recommendation, practice, supplementation, dosage, 2000 IU, safety, RCT

## Abstract

Vitamin D deficiency is considered a public health problem due to its worldwide high prevalence and adverse clinical consequences regarding musculoskeletal health. In addition, vitamin D may also be crucial for the prevention of certain extraskeletal diseases. Despite decades of intensive scientific research, several knowledge gaps remain regarding the precise definition of vitamin D deficiency and sufficiency, the health benefits of improving vitamin D status, and the required vitamin D intakes. Consequently, various societies and expert groups have released heterogeneous recommendations on the dosages for vitamin D supplementation. In this brief narrative review, we outline and discuss recent advances regarding the scientific evidence arguing for a daily vitamin D supplementation with 2000 international units (IU) (50 µg) of vitamin D3 to prevent and treat vitamin D deficiency. According to data from randomized controlled trials (RCTs), such a dose may improve some health outcomes and is sufficient to raise and maintain serum 25(OH)D concentrations above 50 nmol/L (20 ng/mL) and above 75 nmol/L (30 ng/mL) in >99% and >90% of the general adult population, respectively. According to large vitamin D RCTs, there are no significant safety concerns in supplementing such a dose for several years, even in individuals with an already sufficient vitamin D status at baseline. A daily vitamin D supplementation with 2000 IU (50 µg) may be considered a simple, effective, and safe dosage to prevent and treat vitamin D deficiency in the adult general population.

## 1. Introduction

Vitamin D deficiency can be regarded as a public health problem because it has a high prevalence and contributes to skeletal diseases, including rickets and osteomalacia, but may also play a role in certain extraskeletal diseases [1,2]. The main source of vitamin D for humans is ultraviolet-B (UV-B) (sunlight) induced vitamin D synthesis from its precursor 7-dehydrocholesterol in the skin, whereas natural food sources of vitamin D (e.g., fish or mushrooms) play only a minor role in overall vitamin D supply. Limited sunlight exposure of the skin, obesity with deposition of vitamin D metabolites in the adipose tissue, and poor nutrition contribute, amongst others, to the high prevalence of vitamin D deficiency [3,4]. Laboratory detection of vitamin D deficiency is based on the measurement of serum concentrations of 25-hydroxyvitamin D (25(OH)D), the vitamin D metabolite that best reflects the overall supply from all different vitamin D sources, and that is the accepted parameter of vitamin D status. Vitamin D itself is considered biologically inactive and is converted to 25(OH)D by enzymes that are mainly located in the liver.

The clinical role of vitamin D is historically based on the fact that vitamin D was discovered as a substance that is capable of preventing and curing rickets, a bone disease with low serum calcium and low serum phosphate, and the widening and delaying of mineralization of growth plates, leading to bone deformation and muscle weakness in children [5,6]. Further investigations established the role of vitamin D as a regulator of calcium (mineral) and bone metabolism. The discovery of vitamin D receptors (VDR) in almost all human tissues and the fact that VDR activation regulates gene expression like classic steroid hormones, including hundreds of vitamin D-regulated genes, provides a sound scientific basis to postulate a potential role of vitamin D not only for skeletal diseases but also for many extraskeletal chronic diseases, including cancer, autoimmune, or infectious diseases [1,7,8,9]. Although there is evidence from meta-analyses of RCTs supporting the notion that vitamin D supplementation may prevent certain extraskeletal outcomes, great controversy remains regarding the precise role of vitamin D in the context of overall human health [1,2,5,10,11,12,13,14,15,16]. In this context, we wish to underscore the efficacy of vitamin D supplementation for some selected clinically relevant outcomes beyond bone health, with a focus on high-quality and up-to-date meta-analyses. Meta-analyses on vitamin D supplementation and all-cause mortality reported inconsistent results with either a moderate, yet statistically significant, reduction of all-cause mortality by vitamin D or no significant effect [14,17,18]. In this context, a recently published meta-analysis of 80 vitamin D RCTs, including 82,210 participants, documented that vitamin D supplementation reduced the risk of all-cause mortality with an odds ratio (OR) (95% confidence interval (CI)) of 0.95 (0.91–0.99) comparing the vitamin D versus the placebo group [18]. A meta-analysis of 14 RCTs with 104,727 participants reported a relative risk (RR) (95% CI) for vitamin D versus placebo regarding cancer mortality of 0.94 (0.86–1.02) that became significant when restricting the analysis to trials with a daily dosing schedule (RR: 0.88; 95% CI: 0.78–0.98) [19]. In a meta-analysis of 46 RCTs with 75,541 participants, the OR (95% CI) for acute respiratory infections in the vitamin D compared to the placebo group was statistically significant with 0.92 (0.86–0.99) [20]. Regarding exacerbations of chronic obstructive pulmonary disease and asthma control, the evidence from recent meta-analyses of RCTs has largely failed to confirm the significant vitamin D effects reported in older publications [15,21,22,23]. Meta-analyses of RCTs do not document any beneficial effect of vitamin D supplementation on cardiovascular outcomes [24,25]. Regarding pregnancy outcomes, the evidence is inconsistent, but it should be noted that a Cochrane article published in 2019 in 22 RCTs in 3725 pregnant women concluded that vitamin D probably reduces the risk of gestational diabetes, pre-eclampsia, low birth weight, and postpartum hemorrhage, but more high-quality trials and an update of this analysis are required [26]. As a complete summary of vitamin D RCTs on non-skeletal health outcomes is beyond the scope of this narrative review, we refer the reader to some other publications on this issue [2,27,28].

In this brief narrative review, we critically appraise current vitamin D guidelines in the context of recently published evidence from large vitamin D RCTs that may, in our opinion, support re-considerations of vitamin D guidelines towards higher dosage recommendations, i.e., 2000 international units (IU) (50 µg) of vitamin D per day, in the general adult population also covering individuals suffering from chronic diseases. For this aim, we start with a comprehensive outline of current vitamin D guidelines and then describe how the results of recent large vitamin D RCTs have provided important new safety data on vitamin D that may alter previous risk-benefit considerations. We then discuss evidence arguing for higher 25(OH)D target levels compared to the rather conservative threshold levels supported by most nutritional vitamin D guidelines.

As we are well aware of the limitations of a narrative review, we considered the Scale for the Assessment of Narrative Review Articles (SANRA) to improve the methodological quality of our work [29]. Regarding the first two topics of SANRA, i.e., “Justification of the article’s importance for the readership” and “Statement of concrete aims or formulations of questions,” we refer to the paragraph above, noting that recent large vitamin D RCTs with 2000 IU (50 µg) of vitamin D have to be critically appraised in the context of current guidelines, as they may have an impact on future guidelines and daily clinical practice. Regarding the third SANRA topic, i.e., “Description of the literature search,” we performed a PubMed search with the following search terms: “(Vitamin D) AND ((RCT) OR (randomized)) AND ((2000 IU) OR (2000 international units) OR (50 µg))” to find relevant articles for our topic and retrieved 750 publications by this search. We addressed the fourth SANRA topic, i.e., “Referencing,” by supporting our key statements with the respective publications (references). The fifth SANRA topic, i.e., “Scientific reasoning,” is considered by our focus on RCTs and meta-analysis data in this work. The sixth SANRA topic, i.e., “Appropriate presentation of data,” is addressed by presenting data of clinically relevant endpoints and including effect sizes for some major findings [29].

## 2. Current Vitamin D Guidelines

Current guidelines for vitamin D intakes are mainly based on the role of vitamin D in musculoskeletal health, particularly regarding the prevention of rickets and osteomalacia [30,31]. The general framework of vitamin D guidelines is to first establish target serum 25(OH)D concentrations that meet the vitamin D requirements and then to calculate the vitamin D intake doses that are needed to achieve these serum 25(OH)D ranges under conditions of minimal to no sunlight-induced vitamin D synthesis (i.e., during winter) and by assuming that intakes of other nutrients are adequate [30,32]. There exists wide agreement that serum 25(OH)D concentrations below 25 to 30 nmol/L (10 to 12 ng/mL) indicate vitamin D deficiency and should be prevented and treated by vitamin D intake. For serum 25(OH)D concentrations from 25–30 nmol/L (10-12 ng/mL) up to 75 nmol/L (30 ng/mL), there is controversy on the threshold for sufficiency with the main scientific debate on whether concentrations ≥ 50 nmol/L (20 ng/mL) or ≥75 nmol/L (30 ng/mL) should be the target 25(OH)D level for vitamin D sufficiency [5,31,33,34]. As a consequence of this debate and owing to the different approaches and uncertainties regarding the dose-response relationship of vitamin D supplementation and its resulting increase in serum 25(OH)D concentrations, there are numerous vitamin D guidelines and expert recommendations published with a wide range of different recommended vitamin D doses [34,35,36]. Nutritional vitamin D guidelines are usually based on vitamin D intake recommendations under conditions of minimal to no sunshine exposure and cover vitamin D supply from all sources, including diet and supplements. In contrast, we refer in the further text, if not otherwise stated, to vitamin D supplement doses and do not consider additional dietary intakes that are usually very low (i.e., below 200 IU (5 µg) for the vast majority of the population).

Regarding the required vitamin D intakes to achieve serum 25(OH)D concentrations of ≥25–30 nmol/L (10–12 ng/mL) and ≥50 nmol/L (20 ng/mL) in 97.5% of the population, it can be assumed that a daily vitamin D supplement with 400 IU (10 µg) and 800 IU (20 µg) of vitamin D, respectively, is sufficient [30,37,38]. Such vitamin D doses ranging from 400 to 800 IU (5 to 10 µg) of vitamin D per day are generally recommended by nutritional vitamin D guidelines [31]. These recommendations were, however, mainly based on White individuals, whereas recent investigations suggest that there may be much higher intakes required in individuals from other ethnicities, and vitamin D requirements may also vary considerably between different regions or continents, suggesting that some populations may require higher doses than previously estimated [4,39,40,41]. For example, one individual participant data (IPD) meta-analysis of vitamin D RCTs in dark-skinned persons (Black or South Asian descent) residing at higher latitudes (i.e., ≥40° N), estimated vitamin D intakes to achieve serum 25(OH)D levels of ≥50 nmol/L (20 ng/mL) in 90%, 95% and 97.5% of the population at 2008, 2364, and 2672 IU (50.2, 59.1 and 66.8 µg), respectively [40]. These data require considerations in updated dosing recommendations for vitamin D and stand in contrast to previous statements that conservative doses of, e.g., 800 IU (20 µg) (or even less) of vitamin D per day meet the vitamin D requirements for almost everyone [31,42]. It should also be stressed that vitamin D RCTs on dose-response relationships of vitamin D intakes and serum 25(OH)D may probably be prone to healthy volunteer bias and not always well resemble the general population that frequently suffers from conditions with a diminished dose-response curve such as obesity [38]. It has also been revealed that relying on summary statistics (e.g., using conventional meta-analyses) usually underestimates vitamin D requirements as opposed to IPD (meta-)analyses that capture the full between-individual variability in the dose-response curve [30,38]. Of note, achieving serum 25(OH)D concentrations of ≥75 nmol/L (30 ng/mL) in the vast majority of the population may require a daily vitamin D supplementation of about 2000 IU (50 µg) [33,35,43].

In this whole discussion on vitamin D dosage recommendations, one major scientific debate is whether serum 25(OH)D ≥ 50 nmol/L (20 ng/mL) or ≥75nmol/L (30 ng/mL) should be the target and whether a general vitamin D supplementation with doses aiming to achieve ≥75 nmol/L (30 ng/mL), i.e., about 2000 IU (50 µg), is safe for the general adult population [33,42,44]. Given that general recommendations for a vitamin D supplementation with 2000 IU (50 µg) per day would shift the whole 25(OH)D distribution of a given population to higher levels and thus increase the risk of harm by vitamin D overdosing for those at the higher end of this distribution, it was argued that there may be a safety concern with such doses [42,45]. In view of recent publications from large vitamin D RCTs supporting the high safety of such doses, we aim to discuss the evidence arguing for the safety of 2000 IU (50 µg) of vitamin D per day and for the target range of ≥75 nmol/L (30 ng/mL) in this work.

## 3. Safety of a Daily Vitamin D Supplementation with 2000 IU (50 µg)

The safety of vitamin D supplementation is discussed in the context of establishing serum 25(OH)D concentrations above which there is a risk of potential harm from vitamin D overdosing. A classic sign of vitamin D toxicity is hypercalcemia, which does usually not occur until serum 25(OH)D concentrations exceed 150 ng/mL (375 nmol/L) and that requires daily vitamin D intakes over long time periods of more than 20,000 IU (500 µg) [45]. The concept of vitamin D toxicity is also based on potential adverse vitamin D effects that may occur well below the threshold for hypercalcemia. Observational studies indicate a U- or J-shaped association of serum 25(OH)D and various health outcomes [42]. In detail, some, but not all, investigations suggested adverse clinical outcomes for individuals with serum 25(OH)D above 125 to 150 nmol/L (50 to 60 ng/mL), which can be achieved by relatively moderate vitamin D doses [42]. When assuming that a general vitamin D supplementation is shifting the whole 25(OH)D distribution of a population to higher levels, there may be a relatively high risk of vitamin D overdosing in those individuals at the higher end of the 25(OH)D distribution at baseline. As a consequence, caution was stressed for vitamin D doses that may well be below the no adverse observed effect level (NOAEL) of 10,000 IU (250 µg) and even below the respective tolerable upper intake level of 4000 IU (100 µg) (calculated as the NOAEL with a safety margin of 2.5 times), but that may potentially lead to serum 25(OH)D concentrations above 125 nmol/L (50 ng/mL) in a few percent of the population [42,45]. Recent RCTs have significantly contributed to more safety data on this issue and have particularly documented the safety of a dose of 2000 IU (50 µg) of vitamin D per day when administered for long periods in relatively unselected general adult populations [43,46,47]. In particular, the VITamin D and OmegA-3 Trial (VITAL), an RCT of 2000 IU (50 µg) of vitamin D in 25,871 older men and women from the US with an intervention period of 5.3 years showed no significant signs of vitamin D toxicity in the intervention group. However, they had relatively high serum 25(OH)D at baseline and were allowed to take vitamin D supplements up to 800 IU (20 µg) per day in addition to the study medication [46]. In detail, after one year of the VITAL trial, participants allocated to vitamin D achieved serum 25(OH)D concentrations of ≥50 nmol/L (20 ng/mL), ≥75 nmol/L (30 ng/mL), and ≥100 nmol/L (40 ng/mL) at 99.4%, 91.9%, and 53%, respectively [43]. Such high serum 25(OH)D concentrations and missing safety concerns in this well-examined cohort strongly support the safety of a daily vitamin D dose of 2000 IU (50 µg). These data must also be interpreted in light of relatively high serum 25(OH)D levels of the study population at baseline, systematic vitamin D food fortification in the US, and vitamin D supplement use in addition to the study medication by almost half of the study population, all contributing to a higher vitamin D status. Therefore, recommendations of 2000 IU (50 µg) of vitamin D per day in other countries/populations may likewise result in a less significant, and thus even safer, vitamin D exposure. In line with this, one meta-analysis in 15 vitamin D RCTs (3150 participants) supplementing ≥2800 IU (70 µg) for at least one year showed no increase in overall total adverse events (RR: 1.05; (95% CI): 0.88–1.24; 1731 participants from 10 trials) nor kidney stones (RR: 1.26; (95% CI): 0.35–4.58; 1336 participants from 5 trials) when comparing the vitamin D versus the placebo group [48]. This later meta-analysis included RCTs in different populations, including, amongst others, patients with heart failure, epilepsy, multiple sclerosis, chronic obstructive pulmonary disease, or lung transplantation [48]. However, a more recent meta-analysis in 22 RCTs including 12,952 participants with a daily vitamin D supplementation of 3200 to 4000 IU (80 to 100 µg) lasting at least 6 months, revealed a RR (95% CI) for hypercalcemia of 2.21 (1.26–3.87), for falls of 1.25 (1.01–1.55), and for hospitalizations of 1.16 (1.01–1.33), when comparing the vitamin D versus the control group, whereas there was no risk difference for hypercalciuria, kidney stones and mortality [49]. Importantly, vitamin D supplementation versus placebo did not increase the risk of hypercalcemia in a meta-analysis of 11 RCTs in 906 chronic kidney disease patients (RR 0.68; 95% CI: 0.39–1.19) [50]. Apart from this, it should be stressed that the high safety and efficacy of vitamin D supplementation has also been documented by RCTs in pregnant and lactating women [26,51,52,53,54].

Despite outlining the high safety of 2000 IU (50 µg) of vitamin D per day, we want to express some words of caution as vitamin D supplementation does indeed have an undeniable potential for adverse effects depending on the dose, the dosing schedule (with increasing risk with intermittent high dose approaches), and for certain groups [49,55,56,57,58]. One major finding in terms of the safety and efficacy of vitamin D is that daily vitamin D supplementation may be superior compared to intermittent bolus dosing of vitamin D [9,59,60,61,62]. Another important safety issue is that some evidence argues that older and diseased individuals may be more prone to adverse effects of vitamin D overdosing. This suggests that considerations regarding vitamin D supplementation doses should also consider the age of the person [49,62]. It should also be mentioned that there exist inherited pathogenic mutations of CYP24A1 (24-hydroxylase) that lead to impaired vitamin D catabolism and, therefore, predispose to hypercalcemia in individuals who are supplemented with vitamin D [58]. These pathogenic mutations of CYP24A1 are very rare but should be considered in the differential diagnosis of hypercalcemia with low parathyroid hormone concentrations. It should also be considered that a dose of 2000 IU (50 µg) of vitamin D is only about 10% of the 20,000 IU (500 µg) of vitamin D that a human body can produce under optimal circumstances due to sunlight-induced vitamin D synthesis in the human skin, an endogenous vitamin D production that is superior in light versus dark-skinned individuals [34,63]. Interestingly, during our evolution, a high vitamin D responsiveness was probably essential for surviving dark winters as it reduced the adverse consequences of vitamin D deficiency [63].

## 4. Evidence Arguing for a Target Serum 25(OH)D Concentration of 75 nmol/L (30 ng/mL)

**Figure 1:**
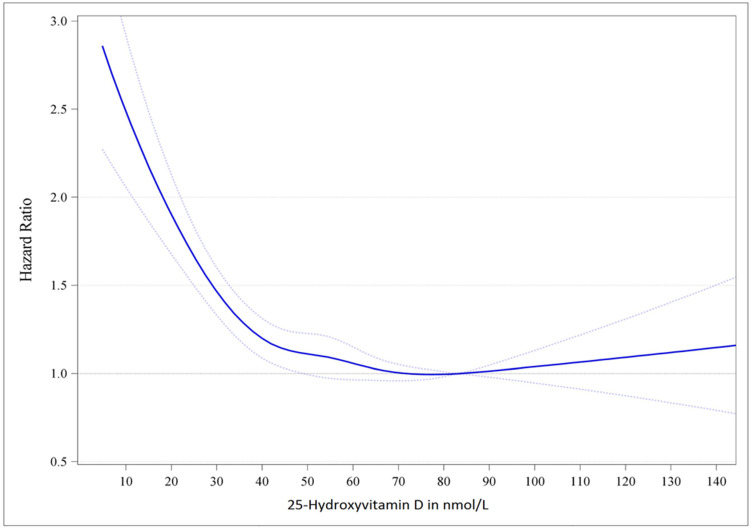
Dose–response trend of hazard ratios of death from all causes by standardized 25-hydroxyvitamin D. Dose–response trend of hazard ratios of all-cause mortality by standardized 25-hydroxyvitamin D were adjusted for age, sex, BMI, and season of blood drawing concentrations. Hazard ratios (blue line with 95% confidence interval as the dotted blue lines) refer to the 25-hydroxyvitamin D concentration of 83.4  nmol/L (i.e., the median 25-hydroxyvitamin D concentration for the group with 25-hydroxyvitamin D concentrations from 75 to 99.99  nmol/L). Reproduced from Ref. [64] under the terms of the CC0 1.0 Universal (CC0 1.0) Public Domain Dedication.

Numerous observational studies have evaluated the risk of adverse health outcomes according to serum 25(OH)D concentrations. Large epidemiological surveys and meta-analyses of observational studies indicate that the lowest mortality risk is present at serum 25(OH)D concentrations slightly above 75 nmol/L (30 ng/mL) [64,65]. When relating serum 25(OH)D to various other health outcomes, it has been documented that for most chronic diseases, optimal serum 25(OH)D concentrations with the lowest risk are above 75 nmol/L (30 ng/mL) [64,65,66,67,68,69]. In detail, one meta-analysis of European cohort studies showed that the lowest mortality risk was detected for serum 25(OH)D concentrations of approximately 78 nmol/L (31 ng/mL) (see Figure 1) [64].

While we cannot definitely claim causality for vitamin D and various extraskeletal diseases, the overall conclusion on the relationship between serum 25(OH)D and various health outcomes based on observational studies is that serum 25(OH)D concentrations above 75 nmol/L (30 ng/mL) are superior to concentrations from 50 to 75 nmol/L (20 to 30 ng/mL) for most clinical endpoints [65]. The exception for this is vitamin D requirements for the prevention of rickets and osteomalacia that are met at lower 25(OH)D concentrations with conservative estimates of about 30 nmol/L (12 ng/mL), although there is also controversy on whether higher levels may be required [5,10]. Thus, the optimal serum 25(OH)D concentration may vary depending on the outcome studied and the population. Some evidence suggests that particularly high 25(OH)D levels may be ideal for certain health outcomes [65,70]. For example, data from the Vitamin D and Type 2 Diabetes (D2d) RCT suggest that based on intra-trial 25(OH)D concentrations, levels of ≥100 nmol/L (40 ng/mL) may be optimal to reduce the risk of diabetes in persons with prediabetes [71]. Serum 25(OH)D concentrations of ≥40 ng/mL (100 nmol/L) or even higher might also be optimal for other health outcomes such as cancer [72,73].

The scientific debate on optimal target concentrations for serum 25(OH)D is, of course, based on risk-benefit considerations, but the argument to not target 75 nmol/L (30 ng/mL) due to safety concerns is, in our opinion, no longer as justified, as it was, and has been acknowledged in previously published vitamin D guidelines [31,42]. Consequently, more weight and attention in this discussion should be paid to the potential extraskeletal health effects of vitamin D, which may require higher 25(OH)D levels than those established for skeletal health. Apart from the above-described epidemiological data that are in line with target concentrations for 25(OH)D of at least 75 nmol/L (30 ng/mL), there are also some findings from RCTs that support relatively high vitamin D doses [19,20,26,27,74,75,76,77,78]. However, regarding vitamin D RCTs, it must be stressed that one of the major limitations of large vitamin D trials was the inclusion of mainly vitamin D-sufficient individuals who were allowed to take vitamin D supplements in addition to the study medication [60,79,80]. Nevertheless, we wish to emphasize that there are several data from RCTs, observational, and molecular studies supporting a beneficial role of vitamin D for various extraskeletal diseases such as cancer, respiratory infections, autoimmune/inflammatory diseases, or diabetes mellitus requiring relatively high vitamin D doses [71,75,76,81,82]. In this context, we are well aware that the high number of RCTs and their post-hoc analyses may probably increase the “false positive” findings, i.e., formally statistically significant results indicating beneficial vitamin D effects; therefore, we must be cautious with the interpretation of such findings [83,84].

An additional argument for a target 25(OH)D level of at least 75 nmol/L (30 ng/mL) is that there is substantial variation regarding the precision of laboratory methods quantifying serum 25(OH)D [85,86]. Targeting a serum concentration of at least 75 nmol/L (30 ng/mL) would, therefore, guarantee that almost all individuals have a 25(OH)D level above 50 nmol/L (20 ng/mL), even if test procedures are used, which overestimate circulating 25(OH)D. The huge individual differences in the response to vitamin D supplementation, evidenced by the molecular effects on vitamin D target genes, could also be considered as supporting higher vitamin D doses to meet the vitamin D requirements of all individuals [87,88,89,90].

We reiterate that in clinical routine, particular attention should be paid to obese individuals who require higher vitamin D doses to increase their serum 25(OH)D concentrations as compared to lean persons and to patients with malabsorption syndromes, such as, e.g., patients suffering from inflammatory bowel diseases, who may also require much higher vitamin D dosages to achieve their serum 25(OH)D target concentrations [91,92]. For example, after two years of supplementing 2000 IU (50 µg) of vitamin D per day in the VITAL trial, the multivariable-adjusted mean serum 25(OH)D concentrations in individuals with a body mass index of <25.0, 25.0–29.9, 30.0–34.9, and ≥35.0 kg/m² were 110, 103, 98, and 92 nmol/L (44.0, 41.2, 39.4 and 37.0 ng/mL), respectively, indicating a highly significant treatment effect interaction by body mass index (*p* < 0.001) [93]. In a meta-analysis of RCTs evaluating the effect of body weight on increases in serum 25(OH)D, the vitamin D dose per body weight explained 34.5% of the variation in 25(OH)D [94]. Patients with inflammatory bowel disease have significantly lower serum 25(OH)D levels compared to matched controls (e.g., 47 versus 62 nmol/L (18.9 versus 25 ng/mL) in one study), in particular during episodes with high disease activity [95,96]. In one vitamin D RCT in 143 patients with inflammatory bowel diseases treated with 25,000 IU (625 µg) once weekly (corresponding to 3571 IU (89 µg) per day), the mean serum 25(OH)D concentrations after 26 weeks was only 81 nmol/L (32.5 ng/mL) in the intervention group [97]. Medications such as antiepileptic drugs that may interfere with vitamin D metabolism may also alter vitamin D status, with one meta-analysis documenting that serum 25(OH)D concentrations were 10 nmol/L (4 ng/mL) lower in patients taking carbamazepine versus controls [98].

Although it is beyond the scope of this present review, there is accumulating evidence and ongoing research on other forms of vitamin D treatment (e.g., calcifediol) or other administration routes (e.g., intramuscularly) that may be useful for certain individuals as reviewed elsewhere [99,100]. Regarding the form of vitamin D that is used for supplementation, we recommend vitamin D3 (cholecalciferol) rather than vitamin D2 (ergocalciferol), as the evidence for treatment efficacy is superior for vitamin D3 and it is also more efficient in increasing serum 25(OH)D concentrations [101,102].

## 5. Practical and Pragmatic Considerations

There are also some practical and pragmatic considerations underpinning the notion that a daily vitamin D supplemental dose of 2000 IU (50 µg) is a reasonable approach to prevent and treat vitamin D deficiency. Using conventional vitamin D doses such as 600 to 800 IU (15 to 20 µg) may, for many individuals, not even be sufficient to achieve the conservative target concentration of at least 50 nmol/L (20 ng/mL) when considering the wide inter-individual dose response according to IPD meta-regression analyses and when taking into account the multiple clinical factors such as obesity, malabsorption syndromes (that may not always be already diagnosed), or medications that impair vitamin D metabolism (e.g., antiepileptic drugs) that all require higher vitamin D doses [35,93,98,103,104]. Clinicians who strictly adhere to nutritional vitamin D guidelines may thus not sufficiently treat their patients with vitamin D supplements when always adhering to conservative dosing regimens not exceeding 800 IU (20 µg) of vitamin D per day [31]. We suggest that clinicians treat vitamin D deficiency of their patients with a supplemental dose that can be either 2000 IU (50 µg) per day as a one-size-fits-all dose or, if they prefer this, that they can tailor the vitamin D dosage according to the patient needs and characteristics as a means of personalized treatment with a dosing range from 800 to 2000 IU (20 to 50 µg). Such an approach may also better meet the preferences of physicians, as we learned from personal unpublished communications from many colleagues. In our own experience, many clinicians have argued that conservative doses such as 800 IU (20 µg) per day were not sufficient to achieve 25(OH)D target levels, and they have thus occasionally proceeded with much higher and potentially harmful vitamin D doses (e.g., >4000 IU (>100 µg) per day) [49,105]. Setting a sufficiently high but not overwhelming vitamin D dosing recommendation with 2000 IU (50 µg) per day may, therefore, also provide helpful guidance in this regard and potentially reduce overdosing of vitamin D. 

The seasonal variation in vitamin D status with higher 25(OH)D levels in summer and lower levels in winter is significantly mitigated by the storage and release of vitamin D metabolites from tissues like the musculature and adipose tissue [3,106,107,108]. Because the large vitamin D RCTs addressing clinical endpoints did not adjust their dose according to season and given that the seasonal variability in serum 25(OH)D may also have a huge interindividual variability with some individuals having almost no seasonal changes in 25(OH)D, we recommend a consistent (identical) vitamin D dose throughout the year [109].

We are well aware that there exist differences between nutritional guidelines to establish dietary nutrient intakes and clinical guidelines that aim to inform physicians. However, without diving into discussions on guideline frameworks and their applications, we wish to stress that accumulating evidence on the safety and efficacy of vitamin D has to be considered in what we, as physicians, but also well-informed non-healthcare experts should be “allowed” to recommend and take as a means to prevent and treat vitamin D deficiency, i.e., 2000 IU (50 µg) of vitamin D per day, without being blamed for deviating from health authority guidelines [33,34,110].

We agree that the best way to achieve a sufficient vitamin D status is a healthy lifestyle, including an optimal diet combined with normal body weight, sufficient physical activity (that may per se mobilize vitamin D metabolites from its body stores), and moderate sunlight exposure [111,112,113]. Improving and optimizing vitamin D status using such an approach should always be prioritized and incorporated into any recommendation. However, we must accept the very high prevalence of vitamin D deficiency and offer simple, safe, and effective approaches to addressing this vitamin D pandemic, i.e., vitamin D supplementation with effective and safe dosages. Apart from this, we are also strong proponents of systematic vitamin D food fortification, but this has not yet been introduced in the majority of countries and may not completely erase vitamin D deficiency [45].

## 6. Conclusions

In this brief narrative review, we have outlined and discussed the evidence arguing for a vitamin D supplementation dose of 2000 IU (50 µg) per day as an efficient and safe approach to prevent and treat vitamin D deficiency (see Table 1).

Considering the evidence outlined and discussed in this brief review, it appears reasonable to consider recommending a daily vitamin D supplement dose of 2000 IU (50 µg) to prevent and treat vitamin D deficiency in the general adult population [39,40,41,43,65]. Such a recommendation is supported by the Endocrine Society clinical practice guideline for evaluating, treating, and preventing vitamin D deficiency, and newer evidence is now reinforcing these older clinical practice recommendations [33]. As we have the impression that the evidence underpinning recommendations for 2000 IU (50 µg) per day is not (yet) well recognized in the scientific literature and the currently published vitamin D guidelines, we drafted this review with the aim that it may serve as a basis for considerations regarding future national health authority guidelines for vitamin D. We are well aware that our work is only a narrative expert review lacking a pre-registered systematic review. We, therefore, have to acknowledge this as a limitation.

Finally, we emphasize that the worldwide prevalence of serum 25(OH)D below 25/30 nmol/L (10/12 ng/mL) and below 50 nmol/L (20 ng/mL) ranges from about 5 to 18% and 24 to 49%, respectively, thereby underscoring the need for actions that aim to reduce the burden of vitamin D deficiency [114]. There exists no “one size fits all” approach for vitamin D supplementation, but as long as individualized approaches, including baseline and follow-up measurements of serum 25(OH)D, are not feasible and cost-effective, we believe that it appears reasonable to recommend a daily dose of vitamin D with 2000 IU (50 µg) when someone asks for advice regarding an effective and safe vitamin D dosage that prevents and treats vitamin D deficiency. A more conservative and personalized approach may suggest a daily vitamin D dose in the range of 800 to 2000 IU (20 to 50 µg) according to the individual needs with characteristics such as very low serum 25(OH)D, obesity, or malabsorption syndromes arguing for the higher end of this dosing range and vice versa.

## Figures and Tables

**Table 1:** Main arguments supporting a vitamin D supplement dose of 2000 IU (50 µg) per day for the prevention and treatment of vitamin D deficiency.

Previous publications and guidelines may have partially underestimated the vitamin D requirements to achieve certain target serum 25(OH)D concentrations.
The high safety of a daily vitamin D supplementation dose of 2000 IU (50 µg) has been well established by recent RCT data documenting this over several years of treatment.
Clinical studies support a serum 25(OH)D concentration of 75 nmol/L (30 ng/mL) and higher as the optimal level.
Some RCT data support clinical extraskeletal benefits of vitamin D supplementation with 2000 (IU) (50 µg) per day.
IU, international units; 25(OH)D, 25-hydroxyvitamin D; RCT, randomized controlled trial

## Data Availability

No new data were created or analyzed in this study. Data sharing is not applicable to this article.
